# A High-Quality Chromosome-Level Genome Assembly of a Snail *Cipangopaludina cathayensis* (Gastropoda: Viviparidae)

**DOI:** 10.3390/genes14071365

**Published:** 2023-06-28

**Authors:** Benhe Ma, Wu Jin, Huiyun Fu, Bing Sun, Su Yang, Xueyan Ma, Haibo Wen, Xiaoping Wu, Haihua Wang, Xiaojuan Cao

**Affiliations:** 1 Jiangxi Fisheries Research Institute, Nanchang 330039, China; mabenhe@126.com (B.M.); jxfuhuiyun@163.com (H.F.); 2 College of Life Science, Nanchang University, Nanchang 330031, China; xpwu@ncu.edu.cn; 3 Key Laboratory of Integrated Rice-Fish Farming Ecology, Ministry of Agriculture and Rural Affairs, Freshwater Fisheries Research Center, Chinese Academy of Fishery Sciences, Wuxi 214081, China; jinw@ffrc.cn (W.J.); maxy@ffrc.cn (X.M.); wenhb@ffrc.cn (H.W.); 4 Wuxi Fisheries College, Nanjing Agricultural University, Wuxi 214128, China; 5 Engineering Research Center of Green Development for Conventional Aquatic Biological Industry in the Yangtze River Economic Belt, Ministry of Education, College of Fisheries, Huazhong Agricultural University, Wuhan 430070, China; sunbing931014@163.com (B.S.); yangsu0407@163.com (S.Y.)

**Keywords:** genome assembly, *Cipangopaludina cathayensis*, comparative genomics

## Abstract

*Cipangopaludina cathayensis* (Gastropoda: Prosobranchia; Mesogastropoda; Viviparidae) is widely distributed in the freshwater habitats of China. It is an economically important snail with high edible and medicinal value. However, the genomic resources and the reference genome of this snail are lacking. In this study, we assembled the first chromosome-level genome of *C. cathayensis*. The preliminary assembly genome was 1.48 Gb in size, with a contig N50 size of 93.49 Mb. The assembled sequences were anchored to nine pseudochromosomes using Hi-C data. The final genome after Hi-C correction was 1.48 Gb, with a contig N50 of 98.49 Mb and scaffold N50 of 195.21 Mb. The anchored rate of the chromosome was 99.99%. A total of 22,702 protein-coding genes were predicted. Phylogenetic analyses indicated that *C. cathayensis* diverged with *Bellamya purificata* approximately 158.10 million years ago. There were 268 expanded and 505 contracted gene families in *C. cathayensis* when compared with its most recent common ancestor. Five putative genes under positive selection in *C. cathayensis* were identified (false discovery rate <0.05). These genome data provide a valuable resource for evolutionary studies of the family Viviparidae, and for the genetic improvement of *C. cathayensis*.

## 1. Introduction

Viviparidae, an almost globally distributed family of freshwater gastropods, belonging to the class Gastropoda, includes a variety of snail species [1]. In China, according to morphological characteristics, Viviparidae are classified into more than 70 species, and divided into nine genera [2]. Among them, *Bellamya* and *Cipangopaludina* are the most speciose [3]. Recently, *B. purificata*, the largest-size species of the genus *Bellamya*, has been deeply studied at the molecular level [4]. Huang et al. [5] performed a transcriptome and proteome analysis and several shell color-related genes/proteins were identified in *B. purificata*. Jin et al. [6] completed genome sequencing and the chromosome-level genome assembly of *B. purificata*. However, molecular genetics studies on the genus *Cipangopaludina* are lacking.

The mudsnail *C. cathayensis*, belonging to the family Viviparidae, order Mesogastropoda, subclass Prosobranchia, class Gastropoda, and phylum Mollusca, is a freshwater snail that is widely distributed in paddy fields, lakes, marshes, rivers, streams, and ponds in China [1,7]. The snail is an edible snail [8]. It has a high nutritional value, containing a variety of essential amino acids, carbohydrates, minerals, and vitamins [9]. It also has a high medicinal value [10]. The Compendium of Materia Medica states that “mudsnails are beneficial to relieve dampness and heat, quench thirst and sober up, facilitate defecation, and cure beriberi and jaundice”. In addition, *C. cathayensis* has many bioactive substances that may be used for tumor and virus suppression [11,12,13].

Due to its high edible and medicinal values, *C. cathayensis* has become a very important aquatic economic animal in China [14]. In recent years, the annual economic value of the “snail rice noodle” has reached more than 10 billion CNY in China. In 2022, it reached more than 50 billion CNY. However, Jin et al. [6] reported that there was a giant gap between the demand and supply of freshwater Viviparidae snails. Therefore, to meet the needs of consumers, the aquaculture and breeding of freshwater Viviparidae snails, including *C. cathayensis*, have become very urgent.

Currently, genome resources for Viviparidae snails are significantly lacking, where only the *B. purificata* genome is available [6]. However, high-quality genome information is very useful for genome-wide selective breeding and economic trait improvement based on the genome editing of *C. cathayensis*. More recently, the rapid development of sequencing technology has made it easier for people to obtain high-quality genomic data [15]. In this study, we assembled a high-quality genome of *C. cathayensis* by using PacBio long-read sequencing and high-throughput chromosome conformation capture (Hi-C) technology. Meanwhile, a comparative genomic analysis was performed to explore the evolution of *C. cathayensis*. The results obtained here are very beneficial to the breeding, aquaculture, and evolution of *C. cathayensis*.

## 2. Materials and Methods

### 2.1. Sample Collection and Sequencing

**Figure 1:**
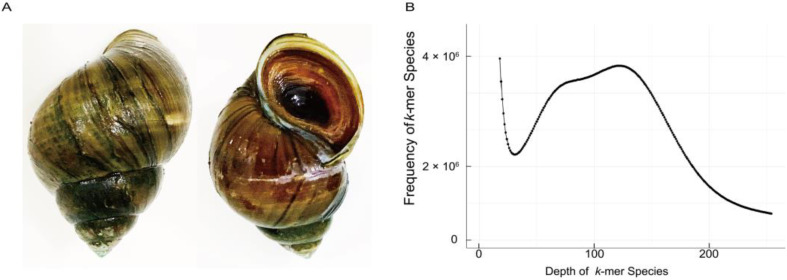
**Physical image and genome survey analysis of *Cipangopaludina cathayensis*.** (**A**) Front (left) and back (right) photographs of *C. cathayensis*. (**B**) Frequency distribution of k-mer depth and k-mer species.

We performed an initial genome assembly to obtain a preliminary estimate of genome size, heterozygosity, and complexity. The genome DNA from the foot of a female specimen of *C. cathayensis* (Figure 1A), collected from the experimental base (28.7298° N, 115.9689° E) of Jiangxi Fisheries Research Institute, was extracted for genome sequencing using a Cetyltrimethylammonium Bromide (CTAB) method. The Blood & Cell Culture DNA Midi Kit (QIAGEN Q13343) was applied for gDNA extraction and purification. The integrity and concentration of the genomic DNA were examined using 1% agarose gel electrophoresis and a Pultton DNA/Protein Analyzer (Plextech).

In the genome survey, the qualified DNA was cut short to 300–500 bp fragments, then terminal repair, addition base A, addition sequence adapter, purification, and PCR amplification were implemented to complete the 350 bp library preparation. Next, we used pair-end sequencing on the MGI DNBSEQ-T7 platform, and fastp with 134.72× coverage (Supplementary Table S1) was used to filter out low-quality reads [16].

Genomic DNA was extracted from the foot of *C. cathayensis* using the QIAamp DNA Mini Kit (QIAGEN). The Agilent 4200 Bioanalyzer (Agilent Technologies, Palo Alto, California) was used to determine the integrity of DNA. Subsequently, g-Tubes (Covaris) and AMPure PB magnetic beads were used for genomic DNA shearing and concentration, respectively. Then, following the method of the Pacific Biosciences SMRT bell template prep kit 1.0, we constructed the SMRT bell libraries. A molecule size of 14–17 kb^2^ was selected for each library by Sage ELF, followed by primer annealing and the binding of SMRT bell templates to polymerases with the DNA Polymerase Binding Kit. The Pacific Bioscience Sequel II platform was selected for sequencing with 43.8× coverage (Supplementary Table S1).

To construct the Hi-C library, we collected the foot of *C. cathayensis* and then treated it with 1–3% formaldehyde at room temperature for 20 min. Subsequently, the gDNA was extracted by the modified CTAB method, and then the restriction enzyme of *Mbo* I and biotinylated residues were used to digest the gDNA and repair the 5′ overhang, respectively. One PE library was constructed with a 300 bp insert size using the Hi-C library preparation protocol. Then, sequencing was performed on Illumina NovaSeq platform and the low-quality reads were filtered out using the fastp software (Version 0.19.5; default parameters) [16]. The Hi-C library was constructed and sequenced with 180.24× coverage (Supplementary Table S1) on the illumina Novaseq 6000 platform to accomplish a chromosome-level genome assembly following the method in [17]. The library was sequenced on the illumina Novaseq 6000 platform.

### 2.2. Genome Size Estimation and Genome Assembly

A k-mer-based method, by making use of Illumina short reads (134.72× coverage) (Supplementary Table S1), was applied to estimate the genome size, heterozygosity, and repeat content in *C. cathayensis* [18]. The PacBio Sequel II sequencing platform was selected for generating long reads with 43.78× coverage and used for genome assembly with HiFiasm software (Version 0.16.1; default parameters) [19].

### 2.3. Hi-C-assisted Chromosome-Level Assembly

The reads were mapped to a non-redundant genome with a bowtie2 (Version 2.4.5) [20]. using default parameters. We removed the redundancy of the preliminarily assembled genome via purge haplotigs (Version 1.0.4) with default parameters [21]. Using Minimap2 (Version 2.23) and SAMtools (Version 1.9) software, we selected the read pairs that were uniquely aligned to the genome at both ends for genome assembly. Subsequently, 3D-DNA with default parameters was used for chromosome-level genome assembly [22] with default settings. The positions and directions of the small contigs were adjusted with Juicebox (VersionVersion 1.11.08) using default parameters [23] manually based on the degree of contig interaction to form the chromosome.

### 2.4. Repeat Annotation, Gene Prediction, and Gene Functional Annotation

Before gene prediction, repeat elements of *C. cathayensis* genome were annotated via homology searches and de novo predictions. RepeatMasker (Version 4.0.9) [24] and Repeat ProteinMask (Version 4.1.0) with default parameters were performed to detect and classify the known repetitive elements by comparing sequences to the Repbase database (https://www.girinst.org/repbase/, accessed on 22 December 2022) [25].

Tandem Repeat Finder (Version 4.09) [26] was executed for the tandem repeat elements detection based on sequence features. Long terminal repeat (LTR)_FINDER (Version 1.0.7) was also applied to ab initio predict the repetitive elements [27], and RepeatModeler (Version 1.0.11) [28] was used for de novo prediction based on libraries de novo constructed with LTR_FINDER (Version 1.0.7) using default parameters, PILER [29], and RepeatScout (Version 1.0.6) [30].

Based on Rfam (Version 14.0) using “cmscan—rfam—nohmmonly evalue 0.01” parameters [31] and miRbase [32] databases, Infernal (Version 1.1) [33] was applied to predict the ribosome RNAs and micro-RNAs, respectively. tRNAscan-SE (Version 1.3.1) with default parameters was used for predicting transfer RNAs [34].

For the prediction of protein-coding genes, homology-based prediction, ab initio prediction, and RNA sequencing (RNA-seq)-based prediction were used. For homology-based annotation, the protein sequences of *Aplysia californica*, *Biomphalaria glabrata*, *Haliotis rubra*, and *Plakobranchus ocellatus* were downloaded from NCBI and aligned to a genome sequence using BLASTN (*e* ≤ 1 × 10^−5^). Homologous sequences were then accurately aligned to corresponding matching proteins using GENEWISE (Version 2.4.0) [35]. The genome sequence was also aligned to a homologous single-copy gene database of Benchmarking Universal Single-Copy Orthologs (BUSCO Version 5.3.1), which was 4analicu_odb10 [36], to find the homologous regions. Maker (Version 2.31.10) with default parameters [37] and HiCESAP (Gooalgene Co., Ltd., Wuhan, China, https://www.gooalgene.com/, https://www.girinst.org/repbase/ accessed on 23 October 2022) were employed to merge all the evidence above and the redundancies were filtered out. De novo genome prediction was performed using AUGUSTUS (Version 3.3.2) and Genscan (Version 1.0). In the meantime, homology annotation was carried out with *A. californica*, *P. ocellatus*, *B. glabrata*, and *H. rubra*, using tblastn (Version 2.11.0+) with an e-value of 0.01. To improve the results of gene prediction, a transcriptome was used for data alignment by utilizing the HISAT2 (Version 2.0.5) software. StringTie (Version 2.1.4) and TransDecoder (Version 5.5.0) were used for transcript prediction and coding region prediction, respectively. Finally, all the annotation results from the above three methods were integrated into a complete annotation file by using Maker (Version 2 2.31.10) with default parameters. The BUSCO (Version 4.1.4) analysis results indicated that it was a high-quality annotation set.

For the gene functionality annotation, some databases of NCBI, InterPro, TrEMBL, SwissProt, and Kyoto Encyclopedia of Genes and Genomes (KEGG) were used throughBlastn and Blastx (*e* ≤ 1 × 10^−5^), and Gene Ontology (GO) annotation was performed using Blast2GO (Version 5.2.5) [38].

### 2.5. Comparative Genomic Analyses and Selection Analysis

OrthoMCL (verison 2.0.9) [39], using “-I 1.5” parameters, was used to detect orthologous groups by retrieving the protein sequence of *Achatina fulica* (PRJNA511624), *B. purificata* (PRJNA818874), *B. glabrata* (ASM45736v1), *Crassostrea gigas* (GCF_902806645.1), *Elysia chlorotica* (GCA_003991915.1), *Lingula anatine* (GCF_001039355.2), *Lottia gigantean* (GCF_000327385.1), *Mytilus galloprovincialis* (GCA_900618805.1), *Patinopecten yessoensis* (GCF_002113885.1), and *Pomacea canaliculata* (GCF_003073045.1). The single-copy orthologous genes shared by all species were multiple-aligned using MUSCLE (Version 5) [40] with default parameters. A phylogenetic tree was constructed using RaxML (Version 8.2.12) [41] based on multiple sequence alignment using “-f a -N 100 m GTRGAMMA” parameters. The divergence time was evaluated using the MCMCTree program of the PAML package using “clock = 3; model = 0” parameters [42].

Phylogenetic relationships were reconstructed from 11 species by using single-copy orthologues. Moreover, gene expansion and contraction analyses were conducted using I (Version 4.0), using default parameters [43]. CAFE simulates gene gains and losses in user-specified phylogenetic trees by birth and death processes. It can calculate the transfer rate of gene family size from parent to child nodes, and infer the gene family size of ancestral species. The gene family size distributions were generated using this model. It can provide a basis for assessing the significance of the observed differences in family size between taxa. We estimated differences across the whole tree using a single birth/death parameter. The significant genes were identified by setting the cutoff *p*-value to 0.05. We performed GO and KEGG enrichment analyses for a better understanding of the biological functions of these genes and the genes of *C. cathayensis* with GO and KEGG annotations used as the background values, respectively. Terms with an enrichment-adjusted *p* value ≤ 0.05 were chosen for further analysis.

The program CODEML (Version4.9) using “branch-site model:A:model = 2, NSsites = 2, fix_omega = 1, omega = 1.0, model = 2, NSsites = 2, fix_omega = 0” parameters of PAML was used for positive selection gene (PSG) identification, and PSGs were also chosen for enrichment analysis. We used the CodeML module in PAML to detect positive selection pressures acting on protein-coding sequences. Firstly, genes were selected from the single-copy gene families. Then, multiple sequence alignment of the gene protein sequences from each single-copy gene family was performed using MAFFT software, and, after that, the results were reversed to the multiple sequence alignment results of CDS. The target species was the foreground branch, and the other species was the background branch. Based on two models, including Model A (assuming the foreground branch ω was under positive selection, ω > 1) and null mode (no sites were allowed to have ω values greater than 1), the likelihood values were calculated, respectively. The likelihood ratio tests (LRTs) were performed on the above likelihood values using the chi2 program in PAML, and the significant difference results were obtained after adjusting the *p* value (FDR < 0.05). Based on the Bayes empirical Bayes (BEB) method, the posterior probability of a site was obtained, which was considered as being positively selected (significant positively selected genes were generally more than 0.95).

## 3. Results and Discussion

### 3.1. Initial Characterization of C. cathayensis Genome

A total of 191.31 Gb clean data were obtained for estimating the genome size of *C. cathayensis*. Using k-mers (K = 17) analysis, an estimated genome size of 1409 Mb was obtained (Figure 1B). Supplementary Table S1 shows the sequencing data information of the *C. cathayensis* genome.

### 3.2. Genome Assembly and Assessment

The 64.84 Gb long reads (Supplementary Table S1) were assembled using HiFiasm (Version 0.16.1) with default parameters, followed by polishing; after that, the redundancy and haplotigs were eliminated, which produced an assembly that was 1.48 Gb in size (Supplementary Table S2). The length of the genome was in accordance with the one estimated using K-mer analysis. The total number of contigs was 40, and N50 reached 98.49 Mb. The genome sizes of other gastropod species including *B. purificata*, *P. canaliculata*, *B. glabrata*, *A. fulica* and *L. gigantean* are between 359 Mb and 2.12 Gb [6,44,45,46,47], which may mean that they drive different genetic mechanisms.

**Figure 2:**
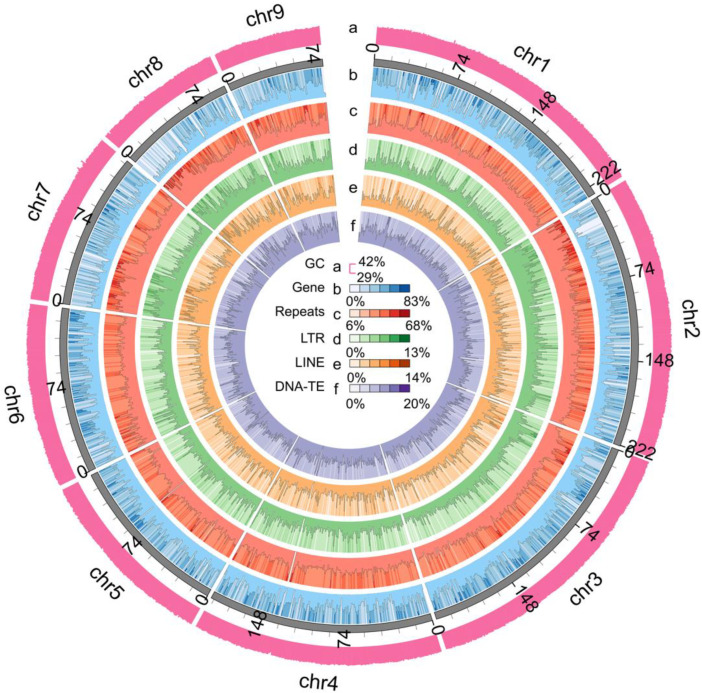
**Genome characteristics of *Cipangopaludina cathayensis*.** From the outer circle to the inner circle, (**a**) GC content of the genome, (**b**) gene distribution, (**c**) repeats, (**d**) long terminal repeat (LTI (**e**) long interspersed nuclear elements (LINE), (**f**) DNA-TE. The height of the bar is proportional to the number of items mapped to each genomic position.

Several genome pieces with a step size of 1 kb were randomly selected and mapped to the NT database (Nucleotide Sequence database), and more than 80% of these pieces could be aligned to the genomes of several shellfish. The BUSCO analysis showed that 94.65% of the complete BUSCO genes (the number was 903) were found in this assembly, including 94.03% for complete and single-copy BUSCO (the number was 897) and 0.63% for complete and duplicated BUSCO (the number was 6) (Supplementary Table S3). A Circos plot of the assessment is shown in Figure 2. These results indicate that the genome of *C. cathayensis* was assembled with high quality.

**Figure 3:**
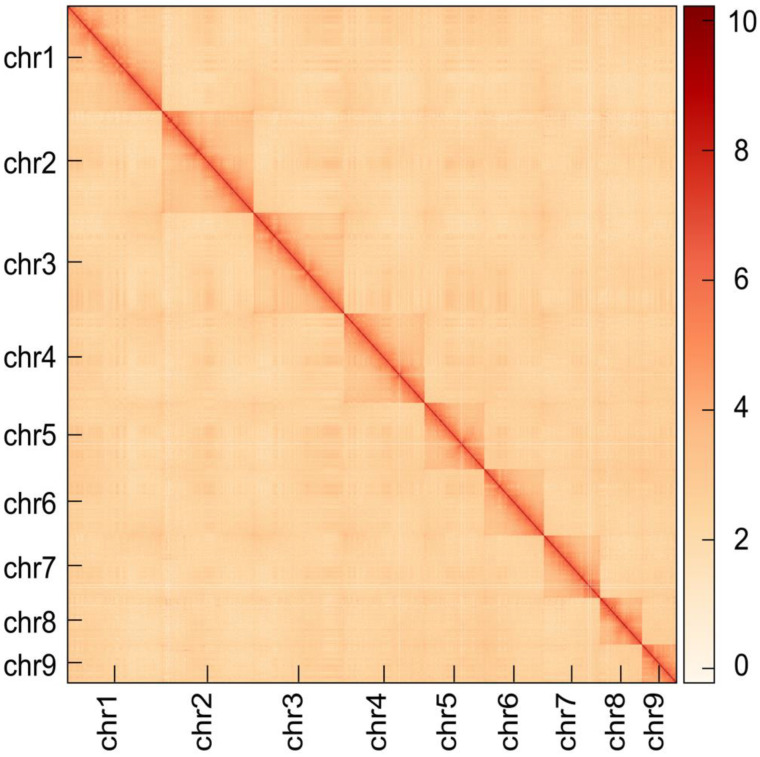
**Genome-wide Hi-C heatmap of *Cipangopaludina cathayensis*.** The blocks represent the nine pseudochromosomes. The color bar illuminated the contact density from white (**low**) to red (**high**).

With the application of the LACHESIS software (Version 0.1.19), 266.94 Gb clean data were used and 99.99% of assembled sequences were anchored into nine pseudochromosomes (Supplementary Table S4). The nine pseudochromosomes can be clearly distinguished from the Hi-C heatmap and the internal interaction was very intense (Figure 3). Moreover, the Hi-C heatmap shows the same results as its karyotypes of *C. cathayensis* (Heude; 2n = 18), and the centromere region revealed by its C-banding is generally a high repeat region in the genome [48]. These results indicated that the nine pseudochromosomes had a high anchoring quality. However, the sex chromosomes are currently unknown, and a subsequent analysis will be conducted through resequencing methods. Finally, the final assembly resulted in high quality genome of 1.48 Gb, with a contig N50 of 98.49 Mb and a scaffold N50 of 195.21 Mb (Supplementary Table S2), which was good for annotation.

### 3.3. Gene Structure and Function Annotations

A total of 797,589,608 bp repeat sequences were annotated, accounting for 53.83% of the total genome sequence (Supplementary Table S5). The proportion was approximately equal to that of the genome survey, but higher than that of *B. purificata* (47.93%). The predominant repeat elements were repeat DNAs (8.83%), LTR (4.45%), and LINE (4.07%) (Supplementary Table S6). Overall, 22, 702 protein-coding genes were predicted (Supplementary Table S7). The average gene length was 25, 375 bp. A total of 18,576 genes, which accounted for 81.83% of all predicted genes, were annotated (Supplementary Table S8). We successfully annotated 68 miRNAs, 208 tRNAs, 135 rRNAs, and 128 snRNAs for non-coding RNA predictions (Supplementary Table S9). A BUSCO evaluation of the predicted genome annotation revealed that 908 orthologus genes (accounting for 95.2%) were matched, including 904 (94.8%) complete single-copy BUSCOs and 4 (0.4%) complete duplicated BUSCOs (Supplementary Table S10).

### 3.4. Comparative Genomics

A comparative genomic analysis of *C. cathayensis* and ten other mollusk species revealed a total of 15,083 gene families and 92 single-copy genes. In the genome of *C. cathayensis*, a total of 22,702 genes were clustered into 19,316 gene families, including 191 unique families. The average gene number per family in *C. cathayensis* was 1.281. For the genomes of other species, the average gene number per family ranged from 1.205 (*B. purificata*) to 3.994 (*M*. *galloprovincialis*) (Supplementary Table S11).

**Figure 4:**
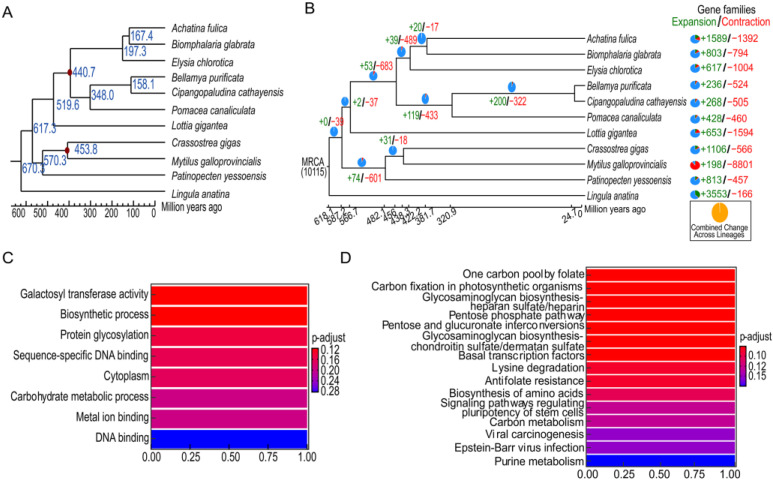
**Comparative genomic analysis.** (**A**) Estimates of species divergence times. The number of node positions represents the divergence time of a species or its ancestors. The red dot is the calibration point. (**B**) Numbers of gene families for expansion and contraction in *C. cathayensis*. The green numbers and the red numbers indicate the number of gene family members that have expanded and contracted during the evolution of the species, respectively. MRCA: Most Recent Common Ancestor. (**C**) GO enrichment of positively selected genes. (**D**) KEGG enrichment of positively selected genes.

The phylogenetic relationships reconstructed from 11 species by using 92 single-copy orthologues confirmed that *B. purificata* is the closest sister lineage of *C. cathayensis* (Figure 4A). Compared to the results of phylogenetic relationships, constructed from Jin et al. [6], we further clarified the evolutionary relationships and positions among the three species of snails (*C. cathayensis*, *B. purificata*, and *P. canaliculata*). The divergence time was estimated using the MCMCTree program, which indicated that the divergence time between *C. cathayensis* and *B. purificata* was estimated to be 158.10 million years ago (Ma), while the divergence time between the former two and *P. canaliculata* was estimated to be 348.0 million years ago (Ma). So, the Viviparidae family was more closely related to the *P. canaliculate* and the viviparous snails may have evolved from oviparous snails (Figure 4A). So far, research on the phylogenetic relationships among snails (Caenogastropoda) remains rare. The complete mitochondrial genomes of eight viviparid snails including *C. ussuriensis*, *C. dianchiensis*, *C. chinensis*, *Viviparus chui*, *Margarya melanioides*, *Margarya monodi*, *B. aeruginosa*, and *B. quadrata* were sequenced to help explore the phylogenetic relationships of caenogastropod snails [3]. It was revealed that some *Cipangopaludina* species (*cathayensis* and *dianchiensis*) should be renamed to be in the genus *Margarya*, and that the *Cipangopaludina* was more closely related to *Margarya* and *Bellamya*. Similarly, our results confirmed the near origin of *Cipangopaludina* and *Bellamya*. Supplementary Table S12 shows that the genome size and GC content of *C. cathayensis* and *B. purificata* were similar.

By comparing the genome of *C. cathayensis* with its most recent common ancestor, we found 268 expanded and 505 contracted gene families in *C. cathayensis* (Figure 4B). Supplementary Table S13 shows the KEGG enrichment analysis results of the expanded genes. These genes (including *slc4a2*, *fgfr, fgfr2*, *fgfr3*, *matk*, *lcp2*, *ctsk*, *sele*, *gstm2* and *gstm3*) were mainly enriched in pathways of salivary secretion, pancreatic secretion, gastric acid secretion, cancer, autophagy-animal, cell adhesion molecules, bile secretion, toll-like receptors, osteoclast differentiation, and platinum drug resistance.

Figure 4C,D show the GO and KEGG enrichment of the positive selective genes, respectively. Five putative genes that appeared to be positively selected in *C. cathayensis* were identified (false discovery rate (FDR) <0.05, Supplementary Table S14). The positively selected genes (include *gart*, *jarid2*, *rpe*, *kmt5a* and *b3galt6*) were mainly enriched in pathways of purine metabolism, carbon metabolism, regulation of the pluripotency of stem cells, biosynthesis of amino acids, antifolate resistance, lysine degradation, glycosaminoglycan biosynthesis (chondroitin sulfate/dermatan sulfate), pentose and glucuronate interconversions, and pentose phosphate and glycosaminoglycan biosynthesis (heparan sulfate).

In *C. cathayensis*, we found an expansion gene *slc4a2* and a positive gene *b3galt6*, which were, respectively, related to digestion [49] and bone development [50]. These findings may suggest that *C. cathayensis* has strong digestive and shell formation abilities in order to adapt well to various environments.

## 4. Conclusions

In conclusion, we assembled the first high-quality, chromosome-level genome of *C. cathayensis*. The assembled genome was 1.48 Gb, including nine chromosomes, with a contig N50 of 98.49 Mb and a scaffold N50 of 195.21 Mb. The genome assembly and annotation are of great importance for the genetic improvement of *C. cathayensis* and lay a strong foundation for evolutionary studies of the family Viviparidae.

## Data Availability

The raw sequencing reads of *C. cathayensis* genome were submitted to NCBI and GSA under PRJNA913660 and CRA009296, respectively. The other data can be obtained by contacting the corresponding authors.

## Supplementary Materials

The following supporting information can be downloaded at https://www.mdpi.com/article/10.3390/genes14071365/s1, Table S1: Statistics for the sequencing data of *C. cathayensis* genome. Table S2: Genome assembly results of *C. cathayensis*. Table S3: BUSCO analysis results of *C. cathayensis* genome. Table S4: Statistics of Hi-C assembly results of *C. cathayensis*. Table S5: Statistics of repetitive sequences in *C. cathayensis* genome. Table S6: Statistics of transposable elements for *C. cathayensis* genome. Table S7: Statistics of gene predictions in *C. cathayensis* genome. Table S8: Summary of functional annotations for predicted genes. Table S9: Statistics of none-coding RNA annotation of *C. cathayensis* genome. Table S10: BUSCO analysis results of *C. cathayensis* genome annotation. Table S11: Statistical results of gene family clustering. Table S12: Comparison of the sequencing data between *C. cathayensis* and *B. purificata*. Table S13: All DEGs were mapped to 83 KEGG pathways, and the number of unigenes in different pathways ranged from 1 to 42. Table S14: List of positive selective genes in *C. cathayensis* (FDR < 0.05).
