# Supplementary material for: A High-Quality Chromosome-Level Genome Assembly of a Snail Cipangopaludina cathayensis (Gastropoda: Viviparidae)
Source: Genes (Basel). 2023 Jun 28;14(7):1365. doi: 10.3390/genes14071365 (PMC10379862; doi:10.3390/genes14071365)
Supplement: Supplementary file 1 [file genes-14-01365-s001.zip › Supplementary Materials.pdf]

Supplementary Tables

Table S1. Statistics for the sequencing data of *Cipangopaludina cathayensis* genome

Type	Platform	Library size (bp)	Raw data(Gb)	Clean data(Gb)	Coverage (×)
MGI	MGI DNBSEQ-T7	350	202.86	191.31	134.72
Hifi	PacBio Sequel II	15k	965	64.84	43.78
Hi-C	MGI DNBSEQ-T7	350	270.03	266.94	180.24
Illumina RNA-Seq	Illumina NovaSeq-6000	350	20.58	20.18	13.63

Table S2. Genome assembly results of *Cipangopaludina cathayensis.*

Mode	Total length (bp)	Total number	Total number (>= 2 kb)	max length (bp)	N50 (bp)	N90 (bp)	GC content (%)
hifiasm	1,493,314,729	77	77	195,581,417	98,494,158	22,719,975	34.58
hifiasm+purge_haplotigs	1,481,564,277	40	40	195,581,417	98,494,158	24,800,090	34.57

Table S3. BUSCO analysis results of *Cipangopaludina cathayensis* genome.

Type	BUSCOs num	Percentage (%)
Complete BUSCOs (C)	903	94.65
Complete and single-copy BUSCOs (S)	897	94.03
Complete and duplicated BUSCOs (D)	6	0.63
Fragmented BUSCOs (F)	9	0.94
Missing BUSCOs (M)	42	4.4
Total BUSCO groups searched	954	100

Table S4. Statistics of Hi-C assembly results of *Cipangopaludina cathayensis*.

	Sequence length (kb)	Sequence number	Contig N50 (kp)	Scaffold N50 (kp)
Draft genome	1,481,564,277	40	98,494,158	98,494,158
Genome after assembly	1,481,416,323	11	98,494,158	195,211,951
Chromosome after assembly	1,481,322,579	9	98,494,158	195,211,951
Free sequence after assembly	93,744	2	75,396	75,396

Table S5. Statistics of repetitive sequences in *Cipangopaludina cathayensis* genome.

Type	Repeat Size (bp)	% of genome
Trf	290,747,731	19.62
Repeatmasker	139,412,236	9.41
Proteinmask	55,542,504	3.75
De novo	469,839,051	31.71
Total	797,589,608	53.83

Table S6. Statistics of transposable elements for *Cipangopaludina cathayensis* genome.

	RepBase TEs		TE Proteins		De novo		Combined TEs	
	Length (bp)	% in genome	Length (bp)	% in genome	Length (bp)	% in genome	Length (bp)	% in genome
DNA Transposable element	87,289,494	5.89	15,304,806	1.03	58,865,912	3.97	130,751,192	8.83
LINE	35,206,647	2.38	23,300,781	1.57	33,482,464	2.26	60,265,603	4.07
SINE	181,402	0.01	0	0.00	1,344,830	0.09	1,448,013	0.10
LTR	31,807,761	2.15	16,949,298	1.14	35,426,263	2.39	65,958,915	4.45
Satellite	5,198,483	0.35	0	0.00	999,543	0.07	6,173,918	0.42
Simple_repeat	0	0.00	0	0.00	167,473	0.01	167,473	0.01
Other	21,420	0.00	0	0.00	0	0.00	21,420	0.00
Unknown	1,388,317	0.09	0	0.00	345,569,012	23.32	346,394,572	23.38
Total	139,412,236	9.41	55,542,504	3.75	469,839,051	31.71	578,440,097	39.04

Table S7. Statistics of gene predictions in *Cipangopaludina cathayensis* genome.

Gene set	Protein coding gene number	Average gene length (bp)	Average CDS length (bp)	Average exon number per gene	Average exon length (bp)	Average intron length (bp)
denovo/Genscan	39,532	22,860	1,244	5.51	225.65	4,790
denovo/AUGUSTUS	63,075	10,221	913.38	4.53	201.67	2,637
homo/*Aplysia californica*	15,502	22,814	1,145	6.26	182.88	4,117
homo/*Biomphalaria glabrata*	17,780	19,394	1,001	5.36	186.56	4,214
homo/*Haliotis rubra*	17,907	24,757	1,196	6.42	186.42	4,351
homo/*Plakobranchus ocellatus*	26,307	12,055	856.32	4.00	214.02	3,731
trans.orf/RNAseq	10,412	41,180	1,823	10.83	481.34	3,659
BUSCO	4,936	26,825	1,719	12.35	139.16	2,211
MAKER	20,503	28,936	1,290	8.10	359.12	3,667
HiCESAP	22,702	25,375	1,533	8.24	351.31	3,105

Table S8. Summary of functional annotations for predicted genes.

Annotated number of predicted genes	Number	Percent (%)
InterPro	15,409	67.88
GO	11,056	48.70
KEGG_ALL	15,599	68.71
KEGG_KO	9,729	42.86
Swissprot	11,875	52.31
TrEMBL	17,649	77.74
TF	1,413	6.22
Pfam	14,419	63.51
NR	17,966	79.14
KOG	11,335	49.93
All annotated	18,576	81.83
Unannotated	4,126	18.17

Table S9. Statistics of none-coding RNA annotation of *Cipangopaludina cathayensis* genome.

Type		Copy	Average length (bp)	Total length (bp)	% of genome
miRNA		68	87	5,946	0.000401
tRNA		208	76	15,764	0.001064
rRNA	rRNA	135	207	27,932	0.001885
	18S	8	1,539	12,313	0.000831
	28S	7	150	1,050	0.000071
	5.8S	7	154	1,078	0.000073
	5S	113	119	13,491	0.000911
snRNA	snRNA	128	145	18,518	0.001250
	CD-box	51	103	5,263	0.000355
	HACA-box	16	232	3,704	0.000250
	splicing	60	157	9,425	0.000636
	scaRNA	1	126	126	0.000009

Table S10. BUSCO analysis results of *Cipangopaludina cathayensis* genome annotation.

Type	Annotation	
	Proteins	Percentage (%)
Complete BUSCOs	908	95.2
Complete Single-Copy BUSCOs	904	94.8
Complete Duplicated BUSCOs	4	0.4
Fragmented BUSCOs	12	1.3
Missing BUSCOs	34	3.5
Total BUSCO groups searched	954	100

Table S11. Statistical results of gene family clustering.

Species	Genes number	Unclustered genes	Genes in families	Family number	Unique families	Unique family genes	Common families	Common family genes	Single copy genes	Average gene number per family
Cipangopaludina cathayensis	22,702	3,386	19,316	15,083	191	565	453	794	92	1.281
Achatina fulica	23,726	4,352	19,374	12,653	386	1,468	453	905	92	1.531
Bellamya purificata	21,476	3,481	17,995	14,934	114	326	453	770	92	1.205
Biomphalaria glabrata	25,539	6,028	19,511	13,419	835	2,821	453	787	92	1.454
Crassostrea gigas	31,371	3,353	28,018	13,844	1,431	7,347	453	991	92	2.024
Elysia chlorotica	23,871	6,764	17,107	13,065	454	1,464	453	690	92	1.309
Lingula anatina	27,055	3,230	23,825	11,686	1,571	6,407	453	1,102	92	2.039
Lottia gigantea	23,818	4,390	19,428	12,714	623	3,344	453	849	92	1.528
Mytilus galloprovincialis	16,208	2,636	13,572	3,398	1,130	5,692	453	675	92	3.994
Patinopecten yessoensis	24,521	3,801	20,720	13,756	807	2,760	453	922	92	1.506
Pomacea canaliculata	21,131	2,262	18,869	12,821	371	1,877	453	764	92	1.472

Table S12. Comparison of the sequencing data between *Cipangopaludina cathayensis* and *Bellamya purificata*

Species	Genome size(Gb)	N50(Gb)	Repeat Size (bp)	GC content (%)
C. cathayensis	1.48Gb	93.49Mb	760.649Mb	34.57
B. purificata	1.01Gb	45.14Mb	482.49 Mb	34.52

Table S13. All DEGs were mapped to 83 KEGG pathways, and the number of unigenes in different pathways ranged from 1 to 42. [xlsx]

Table S14. List of positive selective genes in *Cipangopaludina cathayensis* (FDR <0.05).

ID	Description	GeneID	Gene name	p value	p.adjust	GeneRatio	BgRatio
ko00230	Purine metabolism	Cca0233930.1	GART	0.144192397	0.144192397	1/5	176/5742
ko01200	Carbon metabolism	Cca0242360.1	RPE	0.086558561	0.094427521	1/5	103/5742
ko04550	Signaling pathways regulating pluripotency of stem cells	Cca0099870.1	JARID2	0.078427569	0.094113082	1/5	93/5742
ko01230	Biosynthesis of amino acids	Cca0242360.1	RPE	0.057015717	0.076020956	1/5	67/5742
ko01523	Antifolate resistance	Cca0233930.1	GART	0.043642637	0.065463956	1/5	51/5742
ko00310	Lysine degradation	Cca0048500.1	kmt5a	0.041960391	0.065463956	1/5	49/5742
ko00532	Glycosaminoglycan biosynthesis - chondroitin sulfate / dermatan sulfate	Cca0234620.1	B3GALT6	0.027565497	0.055130995	1/5	32/5742
ko00040	Pentose and glucuronate interconversions	Cca0242360.1	RPE	0.02415345	0.055130995	1/5	28/5742
ko00030	Pentose phosphate pathway	Cca0242360.1	RPE	0.017300621	0.051901864	1/5	20/5742
ko00534	Glycosaminoglycan biosynthesis - heparan sulfate / heparin	Cca0234620.1	B3GALT6	0.015581413	0.051901864	1/5	18/5742
ko00670	One carbon pool by folate	Cca0233930.1	GART	0.051901864	0.051901864	1/5	17/5742
ko00710	Carbon fixation in photosynthetic organisms	Cca0242360.1	RPE	0.051901864	0.051901864	1/5	17/5742
